# Vitamin B3-Based Biologically Active Compounds as Inhibitors of Human Cholinesterases

**DOI:** 10.3390/ijms21218088

**Published:** 2020-10-29

**Authors:** Antonio Zandona, Gabriela Lihtar, Nikola Maraković, Katarina Miš, Valentina Bušić, Dajana Gašo-Sokač, Sergej Pirkmajer, Maja Katalinić

**Affiliations:** 1 Institute for Medical Research and Occupational Health, Ksaverska c. 2, HR-10001 Zagreb, Croatia; azandona@imi.hr (A.Z.); gabrielalihtar@gmail.com (G.L.); nmarakovic@imi.hr (N.M.); 2 Institute of Pathophysiology, Faculty of Medicine, University of Ljubljana, Zaloska 4, SLO-1001 Ljubljana, Slovenia; katarina.mis@mf.uni-lj.si (K.M.); sergej.pirkmajer@mf.uni-lj.si (S.P.); 3 Faculty of Food Technology Osijek, Josip Juraj Strossmayer University of Osijek, Kuhačeva 20, HR-31000 Osijek, Croatia; valentina.busic@ptfos.hr (V.B.); dajana.gaso@ptfos.hr (D.G.-S.)

**Keywords:** AChE, BChE, neurodegenerative, Alzheimer’s, nicotinamide, cytotoxicity

## Abstract

We evaluated the potential of nine vitamin B3 scaffold-based derivatives as acetylcholinesterase (AChE) and butyrylcholinesterase (BChE) inhibitors, as a starting point for the development of novel drugs for treating disorders with cholinergic neurotransmission-linked pathology. As the results indicate, all compounds reversibly inhibited both enzymes in the micromolar range pointing to the preference of AChE over BChE for binding the tested derivatives. Molecular docking studies revealed the importance of interactions with AChE active site residues Tyr337 and Tyr124, which dictated most of the observed differences. The most potent inhibitor of both enzymes with *K*_i_ of 4 μM for AChE and 8 μM for BChE was the nicotinamide derivative 1-(4′-phenylphenacyl)-3-carbamoylpyridinium bromide. Such a result places it within the range of several currently studied novel cholinesterase inhibitors. Cytotoxicity profiling did not classify this compound as highly toxic, but the induced effects on cells should not be neglected in any future detailed studies and when considering this scaffold for drug development.

## 1. Introduction

The therapeutic approach for treating various neurodegenerative disorders and disorders with cholinergic neurotransmission-linked pathology is currently focused mostly on alleviating their symptoms and progression. One such disease is Alzheimer’s disease (AD), which affects the elderly, and myasthenia gravis, which affects people of any age.

AD is a severe degenerative brain disorder leading to dementia, memory loss and disorders of thinking, behaviour and personality [1,2]. The pathology of the disorder has been well researched and is characterised by the formation of cytotoxic plaques of misfolded amyloid β-peptides, hyperphosphorylation of τ-protein in the cells and by a loss of cholinergic neurotransmission [1,2]. Considering that there is no adequate treatment or drug that would act on the cause of the AD, the inhibition of human cholinesterases (acetylcholinesterase, AChE, EC 3.1.1.7; and butyrylcholinesterase, BChE, EC 3.1.1.8) emerged as an important therapeutic regime [3,4,5,6,7,8]. This is important especially since a diminished level of ACh in the brain is one of the characteristics of AD, so drugs designed as AChE inhibitors prevent ACh from fast degradation and enable longer-lasting neuro signals [9,10]. Even though BChE’s involvement in AD is still under investigation [11,12], it is also capable of hydrolysing ACh and thus diminish the effort of an AChE-acting drug [13,14,15]. Therefore, the use of nonselective cholinesterase inhibitors may be beneficial to AD patients as well [15,16].

In contrast to AD, myasthenia gravis is an autoimmune disease affecting the neuromuscular junction [17]. This disease’s main pathology is characterised by antibodies attacking acetylcholine receptors (AChR), muscle-specific kinase (MUSK) and low-density lipoprotein receptor-related protein 4 (LRP4) or other functionally related molecules in the postsynaptic membrane at the neuromuscular junction [17]. The most affected are the eyes, face and throat muscles and, in the later stage of disease progression, the neck and limb muscles [17,18]. As in the case of AD, no cure exists for myasthenia gravis, but treatment can help relieve signs and symptoms [19]. Again, drugs acting on ChE, that increase the amount of acetylcholine at neuromuscular endplates after motor nerve stimulation, alleviate muscle weakness in all myasthenia gravis patients [19].

Numerous structures have been investigated as scaffolds for potential cholinesterase inhibitors for the treatment of both AD [20,21,22,23,24] and myasthenia gravis [25,26]. To this day, only three AChE-acting drugs have been approved in treating AD symptoms: the benzylpiperidine donepezil [27,28], the tertiary alkaloid galantamine [29] and the carbamate rivastigmine [29,30]. Aminoacridine tacrine as an anti-AChE acting drug was withdrawn from the market due to hepatotoxicity [31,32]. Carbamate pyridostigmine is the preferred drug for treating myasthenia gravis, though several other carbamates are available at the market like neostigmine, and oxalamide ambenonium chloride [33].

Due to many side effects as well as a low number of novel candidates entering the late stage of drug-development pipeline, new compounds of better characteristics are constantly being sought [25,34,35,36].

A newly synthesized group of compounds based on vitamin B3, nicotinamide, was recently described as potentially biologically active against several targets [37,38]. Nicotinamide derivatives have broad pharmacological properties especially as inhibitors of many enzymes involved in the pathogenesis of numerous illnesses [39]. They are investigated as antioxidative [40], anticancer [37], anti-angiogenic [41] and antinociceptive agents [42], but also as drugs for treating patients with neurodegenerative diseases [43].

**Figure 1:**
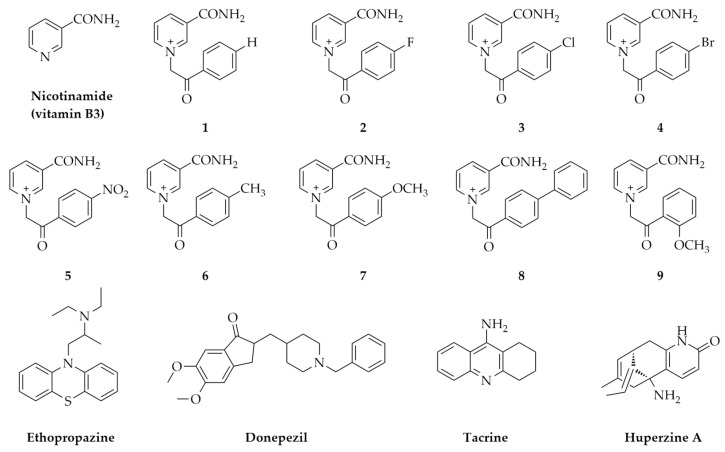
Chemical structures of tested vitamin B3-derivatives **1**-**9** (nicotinamide form) and known ChE acting drugs (reversible inhibitors).

In that sense, we described nine vitamin B3 derivatives (Figure 1) as inhibitors of human AChE and BChE. The synthesis of these compounds has been previously conducted [44,45]. Since these compounds have quaternary nitrogen present in their structure, we performed an in silico analysis of their basic physicochemical characteristics to estimate whether any would possess some characteristic advantageous for acting wider than at the peripheral nervous system (PNS). Furthermore, we performed an in vitro cytotoxicity screening on neural-like (SH-SY5Y) and kidney cells (HEK293) to evaluate their potential to be considered a starting point for novel anti-ChE drugs.

## 2. Results

### 2.1. In Silico Physicochemical Characteristics of Nicotinamide Derivatives

**Figure 2:**
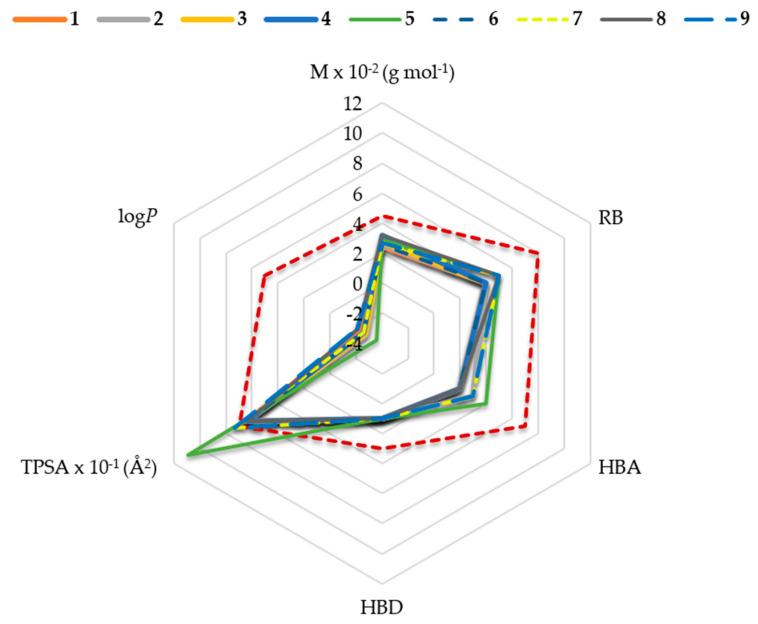
Physicochemical characteristics (molecular weight (M), number of hydrogen bond donors and acceptors (HBD and HBA), number of rotating bonds (RB), lipophilicity (logP) and topological polar surface area (TPSA)) of nicotinamide derivatives in relation to the reference values set for blood–brain barrier (BBB) acting drugs presented here by the red dotted line [46].

According to the in silico determined physicochemical properties, most of the tested nicotinamides possess some characteristics that could work in their favour, like molecular weight (M), number of hydrogen bond donors and acceptors (HBD and HBA) and number of rotating bonds (RB) (Figure 2). However, all compounds have a negative log*P* value, as expected due to the presence of a permanent positive charge. This indicates a low lipophilicity and a low potential to be passively transported to central nervous system (CNS). Furthermore, compounds **5**, **7** and **9** also have a higher value of the topological polar surface area (TPSA) than recommended by the reference values set for a blood–brain barrier (BBB) acting drug [46]. According to in silico calculations, a moderate possibility of a potential passive transport through the BBB was predicted only for compound **8** (Table 1).

### 2.2. Reversible Inhibition of AChE and BChE by Nicotinamide Derivatives

As our results indicate, all of the tested compounds reversibly inhibited both AChE and BChE with the obtained inhibition constants *K*_i_ summarised in Table 2. Structural modifications, in terms of substituent change, influenced both AChE and BChE inhibition. The increase in the effect of the substituent on the side phenyl ring on inhibition constant followed a different order for the two cholinesterases: 4-F < 4-H < 4-NO_2_ < 4-Cl < 2-OCH_3_ < 4-OCH_3_ < 4-Br < 4-Ph ≤ 4-CH_3_ for AChE vs. 4-F < 4-CH_3_ < 4-H < 4-Cl < 4-OCH_3_ < 2-OCH_3_ < 4-NO_2_ < 4-Br < 4-Ph for BChE. When analysing the binding selectivity in terms of the *K*_i_ (AChE) over *K*_i_ (BChE) ratio, all nicotinamide analogues showed minor selectivity in binding but all favoured AChE over BChE (Table 2). The highest selectivity for binding to AChE was observed for compound **6**, which preferred AChE up to 80-times over BChE. Interestingly, both cholinesterases exhibited the highest binding affinity for analogue **8** with the phenyl substituent (4-Ph) at the side ring. However, for AChE this was not a significant difference in inhibition potency when compared to analogue **6**.

### 2.3. Molecular Docking Studies

**Figure 3:**
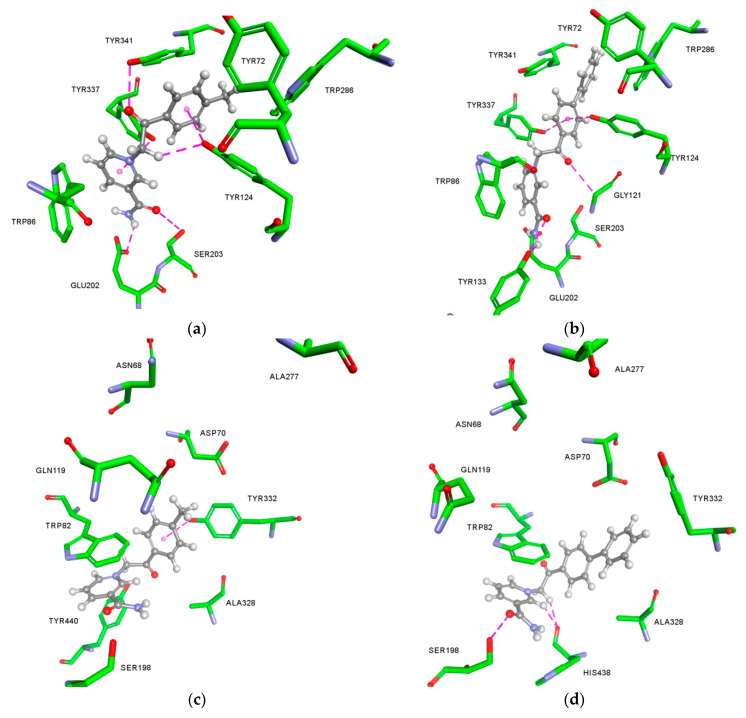
Close-up of AChE and BChE active site from model complexes with compounds **6** (**a**,**c**) and **8** (**b**,**d**). Hydrogen bonds are shown in dashed magenta lines.

The differences in the reversible binding to AChE and BChE of the most potent compounds **6** and **8** were analysed by molecular modelling studies to provide a possible structural explanation (Figure 3). A close-up of the AChE active site shows a similar orientation of **6** and **8** (Figure 3a,b), explaining the similar determined *K*_i_. However, a careful inspection reveals that compound **8** binds in a more favourable way, i.e., more elongated conformation that enables it to protrude closer to the bottom of the active site gorge thus placing phenyl side ring between Tyr337 and Tyr124 and securing a tight fit with two hydrogen bonds from hydroxyl groups of parent residues. In the case of BChE, compound **6** was observed in a similar position as in AChE (Figure 3c). However, in the BChE active site its conformation resulted in only 10 non-covalent interactions established with the amino acid residues compared to the 15 interactions established within AChE (Table A1). This predicted number of interactions within the two enzymes’ active sites supports the observed AChE/BChE selectivity. However, the most potent BChE inhibitor compound **8** seems to be stabilised through an extensive network of interactions with aromatic residues and additionally through hydrogen bonds with Ser198 and His438 of the catalytic triad (Table A1). These aromatic interactions are enabled by a Trp82 side chain rotating to an almost perpendicular position relative to its usual position (Figure 3d). This atypical Trp82 conformation has not been observed previously in published crystallographic structures. However, it is well-known for active site residues in cholinesterase family to be recorded in unprecedented conformations, i.e., in the case of Trp286 and Tyr341 of AChE [51,52]. Therefore, herein, the predicted conformation of Trp82 is not to be resolutely discarded. Furthermore, since the implicit solvent model was used, the absence of water molecules known to be important for docking studies could also explain movement of residues, which likely do not occur physiologically [53]. However, only crystallographic studies will undoubtedly confirm the predicted Trp82 conformation.

### 2.4. Cytotoxicity of Nicotinamide Derivatives

The cytotoxic effect of nicotinamide derivatives on the human neuronal SH-SY5Y and kidney HEK293 cells, in terms of IC_50_ values, is summarised in Table 3. As the results show, four out of nine analogues displayed toxicity within the studied concentration range. The compounds affected both cell lines, though HEK293 cells were more sensitive to the exposure. The lowest IC_50_ value (i.e., the highest toxicity) of about 85 μM was determined for compounds **3** and **4**, with chlorine and bromine substituents present in their structure, respectively.

**Figure 4:**
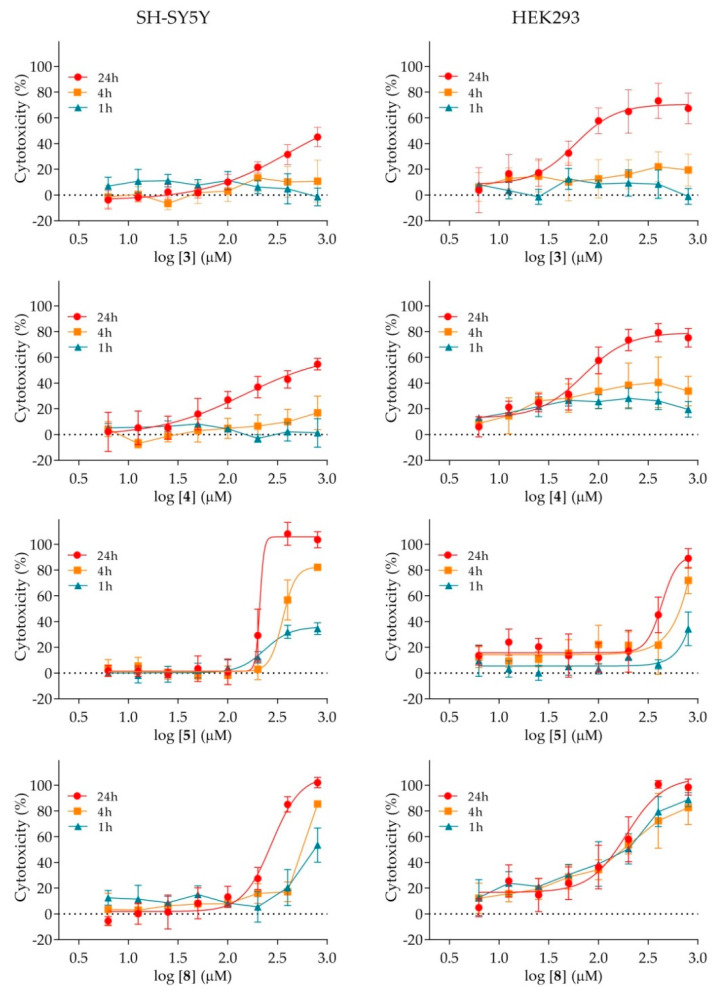
Time and dose-dependent cytotoxicity of selected nicotinamide derivatives on SH-SY5Y and HEK293 cells after 1-, 4- and 24-h treatment. Experimental data was presented as a mean of at least three experiments.

For both cell-lines, a more detailed analysis in terms of time and dose-dependent exposure was performed (Figure 4). As the results reveal, compounds **3**, **4** and **5** displayed a time-dependent mechanism behind the observed toxicity on both cell lines, indicating apoptosis. An exception was observed for compound **8** that seems to affect different cells by a different mechanism. More precisely, while a time-dependent mechanism was observed for SH-SY5Y cells, the similar IC_50_ values determined in the course of time on HEK293 cells indicate that the toxic effect of the compound **8** manifests as soon as the cells are exposed to it, which is an indication of a necrotic-like impact.

### 2.5. Effect of Nicotinamide Derivatives on Intracellular Signalling in HEK293 Cells

**Figure 5:**
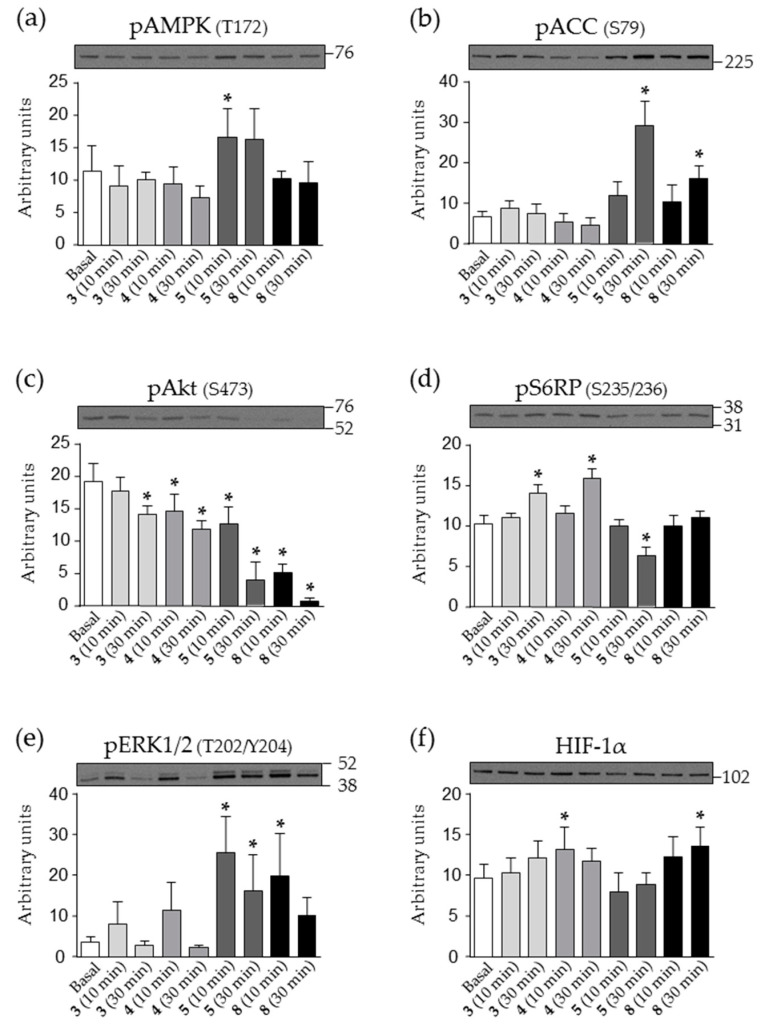
Time-dependent effects of nicotinamide derivatives on intracellular signalling in HEK293 cells. Serum-starved HEK293 cells were treated with 100 μM **3**, **4**, **5** or **8** for 10 or 30 min as indicated. Immunoblotting was used to estimate (**a**) phospho-AMPK (Thr172), (**b**) phospho-ACC (Ser79), (**c**) phospho-Akt (Ser473), (**d**) phospho-S6RP (Ser235/236), (**e**) phospho-ERK1/2 (Thr202/Tyr204), (**f**) HIF-1α. Results are presented as means ± SD (*n* = 6). * *p* < 0.05 vs. Basal (ANOVA followed by Dunnett’s *post hoc* test).

The mechanism of the observed toxic effect was explored further by analysing intracellular signalling in HEK293 cells, which were more sensitive than SH-SY5Y cells. We followed several targets of the most important nicotinamide-linked signalling pathways regulating cell metabolism, growth, proliferation and survival. The obtained results are summarised in Figure 5.

The phosphorylation of AMP-activated protein kinase (AMPK), a cellular energy sensor, and its downstream substrate acetyl-CoA carboxylase (ACC) did not respond to compound **3** or **4** treatment (Figure 5a,b). In contrast, compound **5** markedly increased the phosphorylation of AMPK and ACC indicating AMPK-signalling pathway activation. Furthermore, even though phosphorylation of AMPK was not significantly altered during treatment with **8**, the observed increased phosphorylation of ACC indicated AMPK-signalling pathway activation as well.

All four compounds suppressed phosphorylation of protein kinase B (Akt; Ser473) (Figure 5c), a major kinase downstream of insulin and growth factor receptors, and the effect was markedly pronounced for **5** and **8**. The phosphorylation of S6 ribosomal protein S6 (S6RP, Ser235/236), a downstream effector of the mammalian target of rapamycin (mTOR) pathway, was increased by **3** and **4**, decreased by **5** but at the same time unaltered by compound **8** (Figure 5d). The phosphorylation of extracellular signal-regulated kinase (ERK1/2; Thr202/Tyr204), an important mitogen-activated protein kinase, tended to be increased by all treatments, but the difference reached statistical significance only for compounds **5** and **8** (Figure 5e).

We also measured the abundance of the hypoxia-inducible factor-1α (HIF-1α) (Figure 5f), an oxygen-sensitive transcription factor and a master regulator of oxygen homeostasis. The abundance of HIF-1α was unaltered in cells treated with **5**, but was increased by **4** and **8**. A similar trend was observed for **3**, but the increase was not statistically significant.

## 3. Discussion

Our study included the evaluation of a series of vitamin B3-based, nicotinamide derivatives, with the aim to characterise their inhibition potency towards cholinesterases and the overall therapeutic potential to be considered as a new scaffold for novel drug development.

As our results indicate, all of the tested analogues reversibly inhibited both enzymes in micromolar range. AChE was more susceptible to inhibition, which once again affirmed a contrast in binding affinities between cholinesterases, governed by the amino acid line-up of the active sites. Molecular docking revealed that the interactions formed with the AChE active site narrow part, defined by residues Tyr337 and Tyr124, could be responsible for the differences observed here, especially in the case of compound **6**. Among all of the compounds, the highest inhibition potency for both enzymes was observed for nicotinamide derivative **8** with an additional phenyl ring as a substituent, and this compound stood out as the only one promising for further evaluation as a double ChE-acting inhibitor. Namely, it has been shown that the maintaining the right AChE/BChE activity ratio could be of additional help in treatment of ChE-linked neurodegenerative disorders [54]. The obtained *K*_i_ constants for both cholinesterases places it, for example, in the line with several currently investigated AD and myasthenia gravis treatment drug candidates [8,21,25,54,55,56,57,58], and as such warrants future structure refinement studies. Special focus should be given on improving its predicted physicochemical properties, like removing a positive charge, to increase probability for a passive transport through biological barriers if considered as scaffold for CNS acting drugs. Nonetheless, if by any modifications, the propensity to inhibit ChE would be lost; these compounds scaffold could still be considered primarily for drugs for improving cholinergic signalling in PNS in specific disorders such as the myasthenia [17,18,19].

On the other hand, cytotoxicity profiling of nicotinamide derivatives revealed that in doses of 100 μM they could induce events that could lead to cell death. These responses likely arise from modulation of nicotinamide-linked pathways such as AMPK-, Akt- and mTOR signalling that have an important role in multiple cellular functions [59,60]. What we observed in HEK293 cells treated with compounds **5** and **8** has also been demonstrated in previous studies where nicotinamide derivative NAD, activated AMPK and suppressed Akt and mTOR signalling in kidney mesangial cells [61]. Indeed, consistent with AMPK activation and Akt suppression, the phosphorylation of S6RP as another target, was suppressed by **5** indicating reduced activity of the mTOR pathway. In contrast, compounds **3** and **4** induced phosphorylation of S6RP despite suppressing Akt, indicating that present substituents of such analogues modulate biological activity. Furthermore, while **3** and **4** tended to transiently increase phosphorylation of ERK1/2, compounds **5** and **8** induced a marked increase in its phosphorylation. The transcription factor HIF-1α, with an important role in cellular response to systemic oxygen levels [62], was upregulated by **4** and **8**. This result is consistent with previous studies, which showed that some nicotinamide derivatives are inhibitors of fungal succinate dehydrogenases [63,64,65]. The inhibition of succinate dehydrogenase increases succinate levels and suppresses HIF-1α proteolysis [66]. Thus, the inhibition of succinate dehydrogenase might provide a mechanism by which compounds **4** and **8** increased the abundance of HIF-1α in our study and probably other observed effects as well. Nevertheless, the concentration affecting the cells, especially for the potent ChE-inhibitor **8**, was far greater than predicted to be used in practice according to the determined *K*_i_ or in the general aspect of drug administration [35]. However, the observed effects on cells should not be neglected in any future detailed studies of nicotinamide derivatives as new drug scaffolds.

## 4. Materials and Methods

### 4.1. Nicotinamide Derivatives and Their Physicochemical Characteristics In Silico

Nicotinamide compounds (**1**–**9**) were prepared by quaternization reactions of nicotinamide with differently substituted 2 bromoacetophenones as described previously [44]. The quaternization reactions were carried out by conventional synthesis and by microwave (MW) [44]. The same quaternary salts of nicotinamide were also prepared in agreement with principles of green chemistry in choline chloride based deep eutectic solvents (DES) [45]. The structures of synthesized molecules were determined by one- and two dimensional NMR and IR spectroscopy, mass spectrometry and elemental analysis, and were published previously [44].

The compounds were dissolved in DMSO as 100 mM solutions. Further dilutions were made in water. The final DMSO concentration in enzyme activity measurement experiments did not exceed 0.17% with AChE and 0.6% with BChE and did not affect enzyme activity measurements.

SwissADME interface [67] was used to determine in silico physicochemical properties: molecular weight (M), number of hydrogen bond donors and acceptors (HBD and HBA), number of rotating bonds (RB), lipophilicity (log*P*) and topological polar surface area (TPSA). For all these parameters, the program provides recommendations based on existing drugs [46].

ADME Descriptors protocol implemented in Discovery Studio Client v 18.1 (Dassault Systèmes, Vélizy-Villacoublay, France) was used to predict penetration of compounds to CNS. This model predicts blood–brain penetration after oral administration, and it is derived from over 800 compounds that are known to enter the CNS after oral administration [68]. There are four prediction levels within the 95% and 99% confidence ellipsoids: 0 (very high penetrant)—brain-blood ratio greater than 5:1; 1 (high)—brain-blood ratio between 1:1 and 5:1; 2 (medium)—brain-blood ratio between 0.3:1 and 1:1; 3 (low)—brain-blood ratio less than 0.3:1; 4 (undefined). The plot depicts the five levels with logBB (ADMET_BBB) values as follows: very high penetrants (logBB ≥ 0.7); high penetrants (0 ≤ logBB < 0.7), medium penetrants (−0.52 < logBB < 0) and low penetrants (logBB ≤ −0.52). No prediction is made for compounds outside the 95% and 99% confidence ellipsoids (undefined level = 4).

### 4.2. Reagents and Enzymes

Acetylthiocholine (ATCh) and thiol reagent 5,5′-dithiobis(2-nitrobenzoic acid) (DTNB) for enzyme activity measurement were purchased from Sigma-Aldrich (St. Louis, MO, USA). MTS reagent [3-(4,5-dimethylthiazole-2-yl)-2,5-diphenyl tetrazolium bromide] for cytotoxicity assays was purchased from Promega (Madison, WI, USA).

The source of human AChE and BChE were erythrocytes and plasma, respectively, obtained from the blood of a healthy male volunteer and in accordance with the approval by the Ethics Committee of the Institute for Medical Research and Occupational Health. Erythrocytes were isolated from the whole blood and plasma prepared according to the previously publish procedure [69,70,71]. Namely, blood was taken into tubes containing heparin, centrifuged for 20 min at 2500 rpm (+4 °C). Plasma samples were obtained as supernatant, while intact erythrocytes were washed twice with 0.9% sodium chloride and diluted to the original blood volume. The BChE was phenotyped prior to experiments by the standard procedure described earlier by [69]. The determined BChE phenotype was UU (usual). The presence of AChE in plasma or BChE in erythrocytes was neglectable (confirmed experimentally, data not shown) and therefore enabled the use of the same substrate ATCh to assay enzyme activity without any corrections.

### 4.3. AChE and BChE Reversible Inhibition

The enzyme activity was measured in the presence of a compound tested over a broad concentration range ensuring 20‒80% inhibition of the control enzyme activity. The activity was assayed by Ellman’s method [72]. The assay was performed in the 96-well plates on the Infinite M200PRO plate reader (Tecan Austria GmbH, Salzburg, Austria). Each concentration was tested in triplicate or quadruplicate on each plate. The inhibition mixture contained a 0.1 M sodium phosphate buffer pH 7.4, enzyme (AChE or BChE), tested nicotinamide derivate (0.05–3 mM final) and DTNB (0.3 mM final) and finally the ATCh (from 0.05 to 1 mM final) to start the reaction. The measured activity in the presence of compounds was corrected for the non-enzymatic substrate hydrolysis if detected [73]. The inhibition dissociation constants *K*_i_ were evaluated from the effect of substrate concentration on the degree of inhibition according to the Hunter–Downs equation [74] as described previously [75] using Prism 6 software (GraphPad Software, version 6, San Diego, CA, USA).

### 4.4. Molecular Docking

Ligands to be docked in the receptor structures were created with ChemBio3D Ultra 13.0 (PerkinElmer Inc., Waltham, MA, USA) and minimized using the CHARMm force field and Smart Minimizer minimization protocol of Minimize Ligands protocol implemented in Discovery Studio Client v 18.1. (Dassault Systèmes, Vélizy-Villacoublay, France). Flexible Docking protocol [76] was used for docking ligands into a receptor with flexible selected residues with the following steps being performed: receptor conformations calculation with ChiFlex, ligand conformations creation, ligand docking into active protein conformations with LibDock, poses clustering to remove similar ligand poses, protein conformations rebuilding by refining selected protein side-chains in the presence of the rigid ligand with ChiRotor and final ligand refinement using CDOCKER. The crystal structures of free AChE (PDB ID: 4PQE) [77] and BChE (PDB ID: 2PM8) [78] were used as the receptor, while the binding site within the receptor was defined by a sphere (r = 13.0 Å and 13.5 Å, respectively) surrounding the residues that outline the active sites gorge. The following residues were selected as flexible: Trp86, Tyr133, Trp286, Tyr337, Tyr341 and Tyr449 in the case of AChE and Asp70, Trp82, Tyr128, Leu286, Val288 and Tyr332 in case of BChE [79,80,81]. Details on the rest of the parameters from the Flexible Docking protocol are given in the Appendix section of this paper.

### 4.5. Cytotoxicity Assay

Human neuroblastoma SH-SY5Y (ECACC 94030304), and human embryo kidney HEK293 (ECACC 85120602) used to evaluate the in vitro dose and time dependent cytotoxicity, were obtained from The European Collection of Authenticated Cell Cultures. SH-SY5Y were grown in DMEM F12 (Sigma-Aldrich, Steinheim, Germany) supplemented with 15% (*v/v*) fetal bovine serum (Sigma-Aldrich, Steinheim, Germany), 1% (*v/v*) non-essential amino acids (NEAA, Sigma-Aldrich, Steinheim, Germany) and 1% (*v/v*) antibiotics solution Penicilin-Streptomycin (Sigma-Aldrich, Steinheim, Germany) while HEK293 were grown in EMEM (Sigma-Aldrich, Steinheim, Germany) supplemented with 10% (*v/v*) fetal bovine serum (Sigma-Aldrich, Steinheim, Germany), 1% (*v/v*) non-essential amino acids (NEAA, Sigma-Aldrich, Steinheim, Germany) and 1% (*v/v*) antibiotics solution Penicilin-Streptomycin (Sigma-Aldrich, Steinheim, Germany).

After cultivation, the cells were detached using 0.25% Trypsin/EDTA solution (Sigma-Aldrich, St. Louis, MO, USA), re-suspended and seeded at density of 20,000 cells/well in 96-well plates for experiments. The assay in 96-well was performed in 120 µL/well media volume. The cells were exposed to the tested compounds for 1, 4 or 24 h in a concentration range from 6.25–800 µM. After incubation at 37 °C in a 5% CO2 atmosphere, the cells were washed with phosphate-buffered saline (PBS, 137 mM NaCl, 2.7 mM KCl, 10 mM Na_2_HPO_4_, 1.8 mM KH_2_PO_4_, pH 7.4) and the cytotoxicity profile was determined measuring the succinate dehydrogenase mitochondrial activity of living cells by MTS detection reagent assay [82]. The procedure followed a previously described protocol [83] and the manufacturer’s recommendations. Total percentage of DMSO in cytotoxicity assay was 0.8% and did not influence cell viability. Data was evaluated from at least two or three experiments (each in duplicate or triplicate) using IC_50_ nonlinear fit equation predefined in Prism6 software and presented as percentage of inhibition to control untreated cells.

### 4.6. Analysis of Intracellular Signalling by Western Blot (WB)

Intracellular signalling in the presence of the selected nicotinamide derivative was evaluated on HEK293 cells. To enhance attachment of HEK293 cells, 12-well plates were aseptically pre-coated with poly-lysin (50 µg/mL, 0.5 mL/well). After 1-h incubation, the solution was removed, surface rinsed with sterile demineralized water and allowed to dry for 1 h. HEK293 cells were seeded at a density of 300,000 cells/well in DMEM (1 g/L glucose, Gibco, Paisley, UK) supplemented with 10% (*v/v*) FBS (Gibco, Paisley, UK) and 1% (*v/v*) Pen-Strep (Gibco, Paisley, UK). The next day (after 20 h) the medium was replaced with DMEM without additions. The cells were serum-starved for 4 h. During the last 10 and 30 min, the cells were treated with 100 μM **3**, **4**, **5** or **8**. Basal (control) samples were treated with vehicle (DMSO) for 30 min. At the end of the experiment, cells were washed with ice-cold PBS and lysed in Laemmli buffer (62.5 mM Tris-HCl (pH 6.8), 2% (*w/v*) sodium dodecyl sulfate (SDS), 10% (*w/v*) glycerol, 5% (*v/v*) 2-mercaptoethanol, 0.002% (*w/v*) bromophenol blue). The proteins were resolved with SDS-PAGE (precasted 4–12% polyacrylamide gels, Bio-Rad, CA, USA) and transferred to the PVDF membrane (Merck Millipore, Darmstadt, Germany) with wet electrotransfer using Criterion system and Bis-Tris gradient gels (Bio-rad, CA, USA). After the transfer, the membranes were stained with Ponceau S (0.1% (*w/v*) in 5% (*v/v*) acetic acid) to evaluate uniformity of sample loading and efficiency of transfer. The membranes were then blocked with 7.5% (*w/v*) dry milk in Tris-buffered saline with Tween 20 (TBST: 20 mM Tris, 150 mM NaCl, 0.02% (*v/v*) Tween 20, pH 7.5) for 1 h at room temperature. After blocking, the membranes were incubated with the primary antibody in the primary antibody buffer (20 mM Tris, 150 mM NaCl, pH 7.5, 0.1% (*w/v*) BSA and 0.1% (*w/v*) sodium azide) overnight at 4 °C and then with the secondary antibody-horseradish peroxidase conjugate (Bio-Rad, CA, USA) in TBST with 5% (*w/v*) dry milk for 1 h at room temperature. Finally, the membranes were incubated with enhanced chemiluminescence (ECL) reagent (Bio-Rad, CA, USA) and then immunolabelled proteins were visualized on X-ray films. Films were scanned with GS-800 Densitometer (Bio-Rad, CA, USA) and analysed with Quantity One 1-D 4.6.8 Analysis Software (Bio-Rad, CA, USA). The intensities of individual bands were expressed in arbitrary units relative to the total intensity of all the bands. Target proteins were detected using primary antibodies against phospho-Akt (Ser473, Cell Signaling #4060, Netherlands) phospho-ERK1/2 (Thr202/Tyr204, Cell Signaling #4370, Netherlands), phospho-S6 ribosomal protein (Ser235/236, Cell Signaling #2211, Netherlands), phospho-AMPK (Thr172, Cell Signaling #2535, Netherlands), phospho-acetyl-CoA carboxylase (Ser79, Cell Signaling #3661, Netherlands) and HIF-1α (Novus Biologicals NB100–449, CO, USA).

## 5. Conclusions

The tested vitamin B3-derivatives reversibly inhibited both cholinesterases in the micromolar range and the effect was dependent on the substituent present on the side ring of the molecule. According to the ratio of inhibition constants, it can be concluded that the tested analogues were more selective inhibitors of AChE. The most potent inhibitors of AChE were nicotinamide derivatives **6** and **8**. Derivative **6** has also been singled out as the most selective inhibitor with up to 80-times higher preference for binding to AChE than BChE. The most potent inhibitor of BChE was the nicotinamide derivative **8**, which indicates that an additional aromatic ring as a substituent on the core structure contributes significantly to the binding in the active site gorge of both enzymes. However, four tested analogues showed a cytotoxic effect on both SH-SY5Y and HEK293 cells. Though the determined IC_50_ values do not classify these compounds as highly toxic, the observed interference with several cell signalling pathways should not be neglected in future considerations. Having this in mind, the obtained results taken together support further studies of vitamin B3-derivatives as inhibitors of ChEs, with an emphasis on AChE and PNS, in the development of more efficient drugs for disorders with cholinergic neurotransmission-linked pathology.

## Figures and Tables

**Table 1:** In silico prediction of tested nicotinamide derivatives (**1**–**9**) passive transport to central nervous system (CNS) through the BBB, according to Discovery Studio Client’s ADMET Descriptors protocol. R denotes substituent and its position on the phenyl ring.

Compound	−R	logBB *	Level **
**1**	4-H	−0.885	3
**2**	4-F	−0.821	3
**3**	4-Cl	−0.679	3
**4**	4-Br	−0.654	3
**5**	4-NO_2_	−1.595	3
**6**	4-CH_3_	−0.735	3
**7**	4-OCH_3_	−1.031	3
**8**	4-Ph	−0.416	2
**9**	2-OCH_3_	−1.031	3

* Very high penetrants logBB ≥ 0.7; High penetrants 0 ≤ logBB < 0.7; Medium penetrants −0.52 < logBB < 0; Low penetrants logBB ≤ −0.52. ** Very high penetrant: level 0; High: level 1; Medium: level 2; Low: level 3.

**Table 2:** Reversible inhibition of AChE and BChE by nicotinamide derivatives (**1**-**9**) and known ChE acting drugs (reversible inhibitors). Dissociation inhibition constants (*K*_i_ ± standard error) were determined from at least three experiments. R denotes substituent and its position on the phenyl ring.

Compound	−R	*K*_i_ ± SE (µM)		*K*_i_ (AChE)/*K*_i_ (BChE)
		AChE	BChE	
**1**	4-H	79 ± 11	232 ± 20	0.34
**2**	4-F	85 ± 15	595 ± 73	0.14
**3**	4-Cl	33 ± 5	224 ± 23	0.15
**4**	4-Br	11 ± 3	47 ± 15	0.23
**5**	4-NO_2_	39 ± 5	145 ± 24	0.27
**6**	4-CH_3_	3 ± 1	242 ± 22	0.01
**7**	4-OCH_3_	19 ± 2	216 ± 34	0.08
**8**	4-Ph	4 ± 0.2	8 ± 1	0.50
**9**	2-OCH_3_	26 ± 4	180 ± 18	0.14
**Ethopropazine ^1^**	-	161	0.16	1010
**Donepezil ^2^**	-	0.0043	2.3	0.0019
**Tacrin ^3^**	-	0.190	0.047	4.04
**Huperizine A ^3^**	-	0.082	74.4	0.0011

^1^*K*_i_ values from [47], ^2^ *K*_i_ values from [48,49] and ^3^ IC_50_ values from [50].

**Table 3:** Cytotoxicity of tested nicotinamide derivatives (**1**–**9**) on SH-SY5Y and HEK293 cell line expressed as IC_50_ values (μM). Experimental data was presented as a mean of at least three experiments with standard error. R denotes substituent and its position on the phenyl ring.

Compound	−R	IC_50_ (μM)	
		SH-SY5Y	HEK293
**1**	4-H	≥800	≤800 ^1^
**2**	4-F	≥800	≥800
**3**	4-Cl	≤800 ^1^	85 ± 1
**4**	4-Br	501 ± 2	83 ± 1
**5**	4-NO_2_	214 ± 5	417 ± 1
**6**	4-CH_3_	≥800	≤800^1^
**7**	4-OCH_3_	≥800	≥800
**8**	4-Ph	257 ± 1	155 ± 1
**9**	2-OCH_3_	≥800	≥800

^1^ At the highest tested concentration of 800 μM experimentally determined cytotoxicity point was between 45–50% so the exact IC_50_ value could not be calculated by the software but is therefore estimated.

## Abbreviations

ACC	acetyl-CoA carboxylase
AChE	acetylcholinesterase
AD	Alzheimer’s disease
Akt	protein kinase B
AMPK	AMP-activated protein kinase
ATCh	acetylthiocholine
BChE	butyrylcholinesterase
DMEM	Dulbecco′s Modified Eagle′s Medium
DTNB	5,5′-dithiobis(2-nitrobenzoic acid
EDTA	ethylenediaminetetraacetic acid
EMEM	Eagle’s Minimum Essential Medium
ERK	extracellular signal-regulated kinase
GAPDH	glyceraldehyde 3-phosphate dehydrogenase
HBA	hydrogen bond acceptors
HBD	hydrogen bond donors
HIF-1α	hypoxia-inducible factor-1α
IC_50_	half maximal inhibitory concentration
logP	lipophilicity
M	molecular weight
mTOR	mammalian target of rapamycin
NAD	nicotinamide adenine dinucleotide
PBS	phosphate buffer saline
RB	number of rotating bonds
S6RP	S6 ribosomal protein
TPSA	topological polar surface area

## Appendix A

### Appendix A.1. Molecular Docking

The rest of the parameters from the Flexible Docking protocol were set as follows: Maximum number of protein conformations processed for ligand docking was set to 100; Minimum angel, i.e., torsion angle cut-off (degrees) to determine if two side-chain chi1 angles are the same or not, was set to 25 [84]; Conformation Method, i.e., algorithm for generating ligand conformations, was set to BEST; Maximum number of conformations to be generated was set to 255; Energy Threshold, i.e., relative energy threshold (kcal/mol) within which conformations of separate isomers are created, was set to 20. Parameters for LibDock ligand docking were set as follows: Number of Hotspots, i.e., number of polar or apolar receptor hotspots for conformer matching, and Max Hits to Save were both set to 100 with Tolerance for a hotspots matching algorithm to dock ligands set to 0.25; Maximum Number of Hits which specifies the maximum number of hits saved for each ligand during hotspots matching before the final pose minimization was set to 100 with Final Score Cutoff, i.e., fraction of the top scoring poses reported set to 0.5; Maximum number of poses to be kept per conformation (i.e., Max Conformation Hits) was set to 30 with Maximum number of conformations for each ligand (i.e., Max Start Conformations) set to 1000; Steric Fraction defining number of clashes before the pose-hotspot alignment is terminated was set to 0.10 within the 0.5 Å Final Cluster Radius; maximum values for the Apolar SASA Cutoff and Polar SASA Cutoff values were set to 15.0 Å and 5.0 Å, respectively. Finally, simulated annealing refinement was performed with 2000 heating phase steps during simulated annealing, 5000 steps of the cooling phase, and Heating Target Temperature and Cooling Target Temperature set to 700 K and 310 K, respectively.

### Appendix A.2. The Predicted Non-Bonding Interactions for Modelled Complexes by Molecular Docking

**Table A1:** List of predicted non-bonding interactions for modelled complexes.

Model Complex	Amino Acid	Type of Non-Bonding Interaction
**6**-AChE	Ser203	Hydrogen bond
	Tyr341	Hydrogen bond, π-π stacked, π-alkyl
	Glu202	Hydrogen bond
	Tyr124	Hydrogen bond (x2), π-π T-shaped
	Trp86	π-cation (x2), π-π stacked (x2)
	Tyr337	Hydrogen bond, π-π stacked
	Trp286	π-alkyl
**8**-AChE	Tyr133	Hydrogen bond (x2)
	Glu202	Hydrogen bond
	Gly121	Hydrogen bond
	Trp86	π-cation (x2), π-π stacked (x2)
	Tyr124	Hydrogen bond, π-π T-shaped
	Tyr337	Hydrogen bond, π-π T-shaped
	Trp286	π-π stacked
	Tyr341	π-π T-shaped
**6**-BChE	Trp82	π-cation (x2), π-π stacked (x2), π-π T-shaped
	Tyr332	Hydrogen bond, π-π stacked, π-alkyl
	Tyr440	π-π stacked
	Ala328	π-alkyl
**8**-BChE	Ser198	Hydrogen bond
	His438	Hydrogen bond (x2)
	Trp82	π-cation (x2), π-π stacked (x2), π-π T-shaped (x2)
	Asp70	π-anion
	Ala328	π-alkyl (x2)
